# Measurement of moisture-dependent ion diffusion constants in wood cell wall layers using time-lapse micro X-ray fluorescence microscopy

**DOI:** 10.1038/s41598-020-66916-8

**Published:** 2020-06-18

**Authors:** Joseph E. Jakes, Samuel L. Zelinka, Christopher G. Hunt, Peter Ciesielski, Charles R. Frihart, Daniel Yelle, Leandro Passarini, Sophie-Charlotte Gleber, David Vine, Stefan Vogt

**Affiliations:** 1 Forest Biopolymers Science and Engineering, USDA Forest Service, Forest Products Laboratory, One Gifford Pinchot Drive, Madison, WI 53726 USA; 2 Building and Fire Sciences, USDA Forest Service, Forest Products Laboratory, One Gifford Pinchot Drive, Madison, WI 53726 USA; 3 Biosciences Center, National Renewable Energy Laboratory, 15013 Denver W Pkwy, Golden, CO 80401 USA; 4 X-ray Science Division, Advanced Photon Source, Argonne National Laboratory, 9700 S. Cass Avenue, Lemont, IL 60439 USA

**Keywords:** Polymer characterization, Polymers, Renewable energy, Plant sciences

## Abstract

Our future bioeconomy depends on increased utilization of renewable lignocellulosic biomass. Controlling the diffusion of chemicals, such as inorganic ions, within secondary plant cell walls is central to many biomass applications. However, insufficient understanding of intra-cell-wall diffusion within secondary plant cell walls is hindering the advancement of many lignocellulosic biomass applications. In this work, X-ray fluorescence microscopy was used to measure diffusion constants of K^+^, Cu^2+^, and Cl^−^ diffusing through loblolly pine (*Pinus taeda*) cell wall layers under 70%, 75%, or 80% relative humidity (RH). Results revealed that diffusion constants increased with RH, the larger Cu^2+^ diffused more slowly than the K^+^, and the Cl^−^ diffusion constant was the same as that for the counter cation, indicating cations and anions diffused together to maintain charge neutrality. Comparison with electrical conductivity measurements showed that conductivity is being controlled by ion mobility over these RH. The results further support that intra-cell-wall diffusion of inorganic ions is a Fickian diffusion process occurring through rubbery amorphous polysaccharides, which contradicts previous assertions that intra-cell-wall diffusion is an aqueous process occurring through water pathways. Researchers can now utilize polymer science approaches to engineer the molecular architecture of lignocellulosic biomass to optimize properties for specific end uses.

## Introduction

Lignocellulosic biomass holds great potential as a renewable source of fuels, chemicals, and materials for a variety of applications^1,2^. Diffusion within woody plant cell walls impacts nearly every application of biomass utilization. In biorefineries, the efficacy of many biomass conversion processes to produce fuels and chemicals is highly dependent on diffusive transport of deconstruction catalysts, such as inorganic ions^3^, acids^4^, and enzymes^5^ into cell walls. Similarly, rapid transport of depolymerization products out of cell walls is desirable to minimize enzyme inhibition and unwanted secondary reactions, especially in the case of thermochemical conversion processes such as fast pyrolysis^6,7^. Cell wall diffusion is also important in the manufacture of wood-based building materials, including treatments of wood with copper-based waterborne wood preservatives^8^, adhesive bonding of wood^9,10^, and chemical modifications of wood^11^. Additionally, wood-based materials are susceptible to damage mechanisms, such as fungal decay and fastener corrosion, that also require diffusion through the cell wall^12^. The most common and most economically destructive forms of decay, brown and white rot^13^, require the diffusion of small compounds into the cell wall prior to enzymatic breakdown^14–19^, as well as diffusion of oligosaccharides out of the wood to sustain the fungus. Fastener corrosion, which typically occurs in wood treated with waterborne wood preservatives, requires diffusion of cupric ions from the wood preservatives to the metal surface, where they are reduced as the fastener is oxidized^20–22^. Recently, wood-based materials are also finding application in green electronics, which rely on ionic mobility in cell walls to store and transport charge^23^. Ion diffusion through wood cell walls has also been shown to be a stimuli-responsive phenomenon, which serves as bioinspiration for the design of new multifunctional smart materials^24^. Advancement of these technologies is limited by an incomplete understanding of the mechanisms and rates of intra-cell-wall diffusion. A more complete, quantitative description of diffusion in plant cell walls will enable optimization of cell wall diffusion processes for specific end uses and accelerate utilization of lignocellulosic biomass to meet our societal needs.

**Figure 1:**
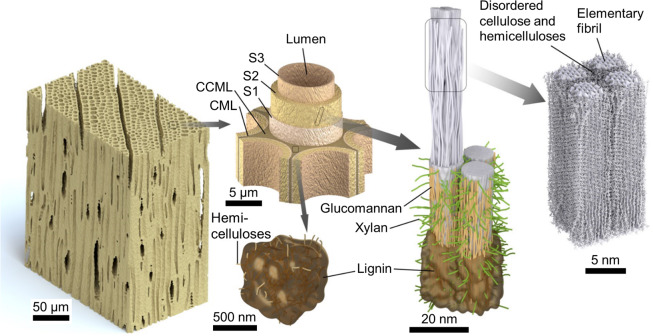
Schematics illustrating the breakdown of softwood from the cellular to nanoscale. Secondary cell walls (S1, S2, and S3) are nanofiber reinforced composites of cellulose fibrils embedded in an organized matrix of amorphous cellulose, hemicelluloses (glucomannan and xylan), and lignin^47^. Cellulose organizes into highly ordered elementary fibrils approximately 3 nm across^83^, which organize into bundles that form microfibrils with amorphous polysaccharides between them^42^. Individual cells are adhered to each other by the compound middle lamella (CML), which is made of an open-cellular hemicelluloses structure encrusted with lignin^49^. At the corners between multiple cells, the large volume of CML is termed the corner CML (CCML).

Lignocellulosic biomass consists of cells that can be thought of as hollow tubes with multilayer walls. The structure of softwood, which is the lignocellulosic biomass used in the study, is schematically depicted in Fig. 1. Although cellular structures vary widely between different types of biomass, the general models of secondary cell wall nanostructures shown in Fig. 1 are representative.

Water content also plays a large role in dictating properties, including intra-cell-wall diffusion^25^, of lignocellulosic biomass. In general, water is classified as being either “bound water”, which is contained within the cell wall material and absorbed to the cell wall polymers, or “free water”, which exists in macroscopic voids, such as the cell lumina or pit chambers, and can be in a vapor, liquid, or solid state. The wood moisture content (MC) is defined as the mass of water divided by the oven-dry wood mass^26^. The MC at which there is the maximum amount of bound water is called the fiber saturation point (FSP) and, depending on how it is measured, is normally found to be between 30% and 40% MC^27–32^. Above the FSP, additional water is free water. As reviewed and discussed by Zelinka and coworkers^33^, previous experimenters also proposed “Type II” or “loosely bound” water in unmodified wood cell walls. However, the observations of these types of water are actually now understood to be artifacts caused by the sample preparations used in those studies^33^. Therefore “Type II” or “loosely bound” water do not likely exist in unmodified wood cell walls.

Recently, it was proposed and experimentally confirmed that intra-cell-wall diffusion of inorganic ions and similar chemical species is a solid diffusion process occurring through pathways formed by interconnecting regions of amorphous polysaccharides that have passed through a moisture-induced glass transition and are in their rubbery state^12,34,35^. In solid amorphous polymers, diffusion of larger chemicals, such as inorganic ions, does not appreciably occur when the polymer is in its glassy state, whereas diffusion does occur when the polymer is in its rubbery state^36–38^. Diffusion is understood to occur in the rubbery state because of the increases in free volume and cooperative motion relaxations that occur as the polymer passes through its glass transition^39–41^. Through a review and analysis of available literature, Jakes and coworkers determined that the *in situ* amorphous polysaccharides glass transitions most likely occurs when the bulk wood is at 10% to 15% MC, which corresponds to 60% to 85% relative humidity (RH) conditions^25^. Proposed interconnecting regions of amorphous polysaccharides in the cell wall nanostructure (Fig. 1) include amorphous cellulose and hemicelluloses between elementary fibrils^42–44^, glucomannan sheaths around microfibrils^45,46^, xylan organized perpendicular to the microfibrils^47,48^, and the limbs in the open-cellular hemicelluloses structure in the CML^49^. This solid polymer diffusion mechanism is in direct contrast to all the previous intra-cell-wall diffusion models where ion transport was assumed to be an aqueous process occurring through interconnecting pathways of free or “loosely bound” water^50–54^.

Despite recent advances in understanding intra-cell-wall diffusion mechanisms, accurate quantification of diffusion is lacking. Quantification of ion diffusion through water-saturated wood cell walls above FSP has been the subject of a few previous studies, but results have varied widely. For example, for copper ions, reported results span five orders of magnitude from 1 × 10^−11^ to 1 × 10^−6^ cm^2^/s^55–59^. Measurements of diffusion through cell walls below FSP are completely lacking.

A major challenge to accurately measuring intra-cell-wall diffusion constants is obtaining time-dependent concentration profiles of the diffusing species in the micron-sized wood cell wall layers. This challenge is overcome here by using a synchrotron X-ray microprobe to make X-ray fluorescence microscopy (XFM) ion maps of locally applied inorganic ions as they diffuse through wood cell wall layers being conditioned at different RH levels. XFM has been shown to have the sensitivity and spatial resolution to accurately map ions in individual S2 and CML^34,60–62^. In this work, time-dependent concentration profiles of diffusing ions were directly measured from time-lapse XFM ion maps obtained *in situ* during diffusion. The diffusion constants were then calculated from the concentration profiles using an analytical model developed based on Fick’s second law for diffusion (see Supplementary Information). Finally, to further validate the diffusion constant measurements and better understand electrical conductivity mechanism in wood, electrical conductivity calculated using the Nernst-Einstein relationship and XFM-based diffusion constants were compared to literature cell wall electrical conductivity measurements^63^.

## Results

**Figure 2:**
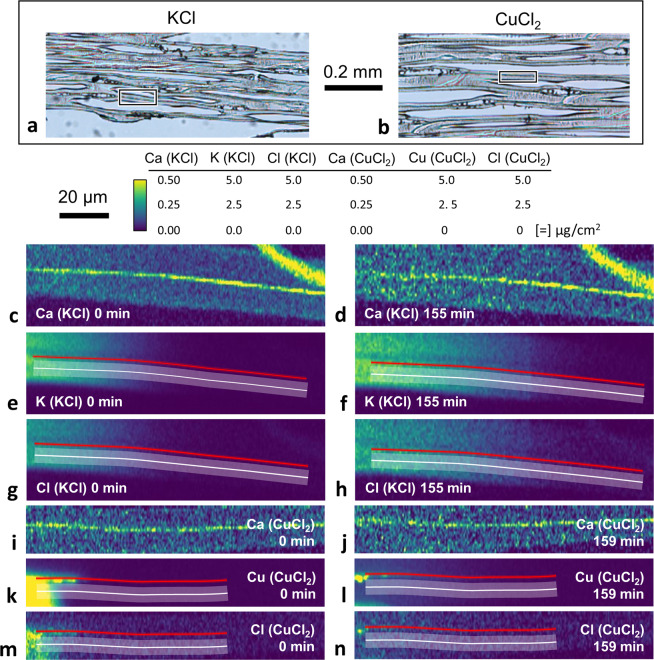
Optical microscopy images of 2-μm-thick sections of loblolly pine used for (**a**) KCl or (**b**) CuCl_2_ diffusion experiments. The rectangles in (**a**,**b**) indicate the cell walls imaged with X-ray fluorescence microscopy (XFM). XFM intensity maps from the first and final images of time-lapse series in cell walls with (**c**–**h**) a locally applied KCl aqueous microdroplet and conditioned at 70% relative humidity (RH), or (**i**–**n**) a locally applied CuCl_2_ aqueous microdroplet and conditioned at 75% RH. The Ca maps are also included to help identify anatomical features in these longitudinal sections. In all Ca maps, the higher intensity line that stretches from left to right is the compound middle lamella (CML). The longitudinal wood orientation is parallel to the CML. The high intensity feature in the upper right-hand corner of the Ca KCl maps was a piece of debris lying on top of the section that did not interfere with the diffusion experiment. The lowest intensity regions in the Ca map were empty lumina. The material with intensities between the CML and lumina was identified as S2 secondary cell wall layer.

For diffusion experiments, 2-μm-thick sections of loblolly pine (*Pinus taeda*) were prepared with tangential-longitudinal orientation (Fig. 2a,b). Inorganic ions were locally applied into the S2 and CML using microdroplets of KCl or CuCl_2_ aqueous solutions. Time-lapse XFM ion maps were then collected while the sections were conditioned inside an *in situ* conditioning chamber held at 70%, 75%, or 80% RH. The RH values were chosen to be above the 60% to 67% RH range in which the onset of diffusion for both K and Cl was observed for similar longitudinal sections in previous studies^34,64^. The six series of time-lapse XFM maps obtained in this study are listed in Table 1. Figure 2 shows first and final salt cation and anion maps from the CuCl_2_ 75% RH (Figs. 2e–h) and a KCl 70% RH (Fig. 2k–n) time-lapse series. The Ca maps (Fig. 2c–j) are also shown because Ca is a naturally occurring inorganic ion in wood and is useful to visualize wood anatomical features in the sections, including empty lumina, S2, and CML. The ions were locally applied on the left-hand side of the maps, and the ion fronts in the S2 and CML were nominally perpendicular to the cell wall longitudinal direction. During diffusion, ion fronts moved to the right in the maps while remaining perpendicular to the longitudinal direction. This observation supported the assumption that diffusion was unidirectional for these experiments in the S2 and CML.

**Table 1:** Time-lapse XFM series for S2 and compound middle lamella (CML) cell wall layers.

Salt	RH (%)	S2		CML	
		Number of images	Total time (min)	Number of images	Total time (min)
KCl	70	6	188	6	188
KCl	70	5	155	5	155
KCl	75	8	159	N/A	N/A
KCl	80	7	65	6	65
CuCl_2_	75	8	159	8	159
CuCl_2_	80	6	69	5	56

**Figure 3:**
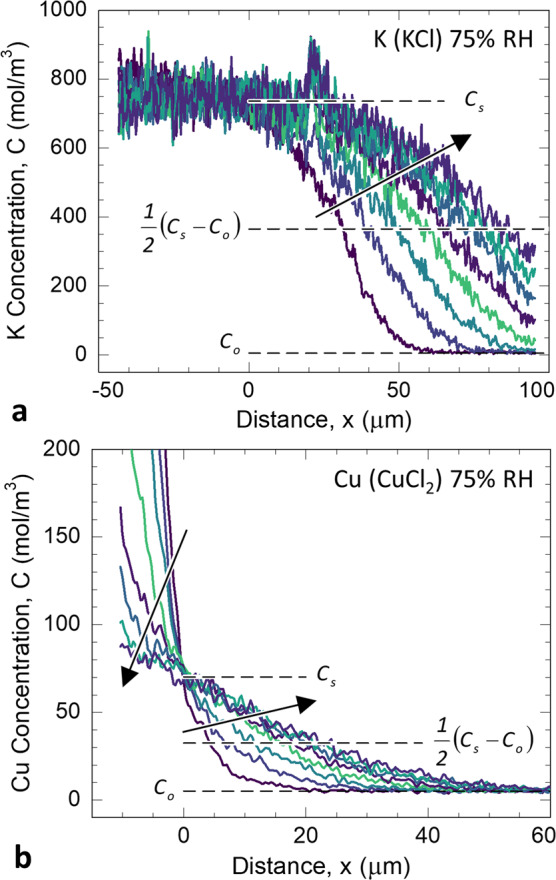
Time-dependent cation concentration (*C*) profiles measured from the S2 cell wall layer in (**a**) KCl 75% RH and (**b**) CuCl_2_ 75% RH specimens as a function of distance (*x*) and time (*t*). In the KCl 70% RH specimen (**a**), the average K *C* remained unchanged up to distance *x* = 0, and then increased with increasing *t* for x > 0. This behavior is indicative of infinite source diffusion. In the CuCl_2_ 75% RH specimen (**b**), with increasing *t* the Cu *C* decreased for *x* < 0 and increased for x > 0. This behavior is indicative of finite source diffusion. For both series the *C* were measured at times 0, 23, 45, 68, 90, 112, 135, and 159 min. Each colored line represents one time, and the arrows point to increasing time. The indicated parameters *C*_o_ and *C*_s_ were used in the diffusion constant analysis developed in the Supplementary Information and are described in the text.

Line segments in the CML (red lines in Fig. 2e–h,k–n) and S2 (broad semi-transparent white lines in Fig. 2e–h,k–n) XFM ion maps were used to extract ion intensity profiles along the diffusing direction. Example molar concentration (*C*) profiles of K and Cu from the S2 are shown in Fig. 3 as a function of distance (*x*) and time (*t*). The XFM map intensity, which had units of µg/cm^2^, was converted to *C* by dividing the XFM map intensity by the ion molar mass and the 2-µm section thickness. The concentration profiles, denoted *C*(*x*,*t*), are plotted such that ions were applied at negative values of *x* and diffusion occurred in the positive *x*-direction. Each salt had distinctively shaped *C*(*x*,*t*). Figure 3a,b illustrate the representative behavior of the diffusing ions applied with KCl and CuCl_2_ solutions, respectively. The anion *C*(*x*,*t*) behavior was similar to its salt cation concentrations that were close to the expected salt stoichiometric ratios.

Independent of RH or type of cell wall layer, the diffusion of K and Cl in KCl-applied sections displayed *C*(*x*,*t*) similar to that displayed in Fig. 3a. The *C*(*x*,*t*) remained unchanged up to a given *x*, which was used to define *x* = 0 for each KCl *C*(*x*,*t*). As *t* increased, the concentration profiles began to increase for *x* > 0. All KCl specimens shared the common feature of an unchanging *C*(*x*,*t*) in the locally applied region up to some value of *x*. This type of diffusion in the KCl sections (Fig. 3a) approximates infinite source diffusion because of the unchanging *C* for *x* < 0. This likely resulted from excess KCl salt maintaining the constant *C* for *x* < 0.

In contrast, all Cu and Cl profiles from CuCl_2_ sections had behavior similar to the *C*(*x*,*t*) in Fig. 3b independent of RH or type of cell wall layer. These *C*(*x*,*t*) had a cross over point, which was used to define *x* = 0. In the CuCl_2_ sections, for *x* > 0 *C* increased with time and for *x* < 0 *C* decreased with time, which is classified as finite source diffusion. The finite source diffusion behavior suggests that there was no excess CuCl_2_ salt maintaining the constant *C* for *x* < 0. The *C*(*x*,*t*) in Fig. 3b corresponds to the XFM intensity maps in Fig. 2i–n. For reference, the defined *x* = 0 location is about 10 µm from the left-hand ends of the line segments drawn in Fig. 2i–n.

**Figure 4:**
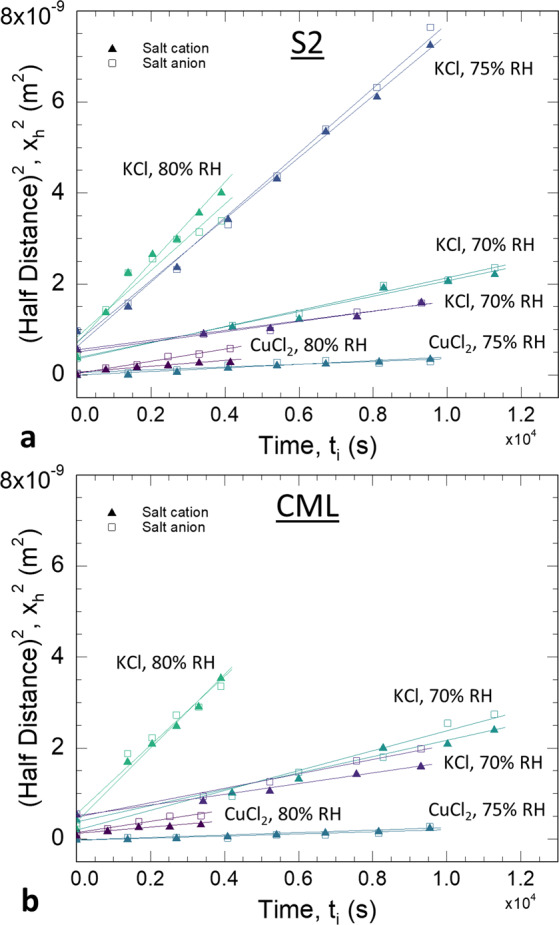
Plots following Eq. 1 used to calculate diffusion constant (*D*) in the (**a**) S2 secondary cell wall and (**b**) compound middle lamella (CML).

Although both infinite and finite source diffusion were observed, the analytical methods developed in the Supplementary Information were applied to obtain diffusion constants (*D*) from both diffusion regimes by employing the relationship

1 $${x}_{\text{h}}^{2}=D{t}_{i}+D{t}_{o}$$

where *t*_i_ is the time after the initial XFM map*, t*_o_ is an unknown time that accounted for the effective time it would have taken for a step function to become the *C*(*x*,*t*_*i*_ = 0) profile, and *x*_h_ is termed the “half-distance” and is the *x* that satisfies *C*(*x*) = 0.5(*C*_s_ + C_o_) for a given *C*(*x*) profile. After calculating 0.5(*C*_s_ + C_o_) for each set of data, as illustrated in Fig. 3, the *x*_h_ was identified from each *C*(*x*) profile. The form of Eq. 1 indicates that a plot of x_h_^2^ vs. *t*_i_ will be a straight line of slope *D*. Plots of x_h_^2^ vs. *t*_i_ for each time-series are shown in Fig. 4. To a good approximation, all these plots were straight lines, which supports the validity of our proposed analysis based on Fick’s second law for one-dimensional diffusion with a constant *D*. Yata and coworkers similarly found straight line behavior between time and the square of depth of penetration for Cu, Zn, or Cr ions diffused into wood cell walls under water-saturated conditions^57,65,66^. Collectively, the data indicate that ionic diffusion may be treated as Fickian in unmodified wood cell walls, at least under the conditions from 70% RH up to water-saturated. Fickian diffusion is also expected because inorganic ion diffusion in wood cell walls occurs through interconnecting regions of rubbery amorphous polysaccharides^35^, and Fickian diffusion is expected in rubbery polymers^67^.

**Figure 5:**
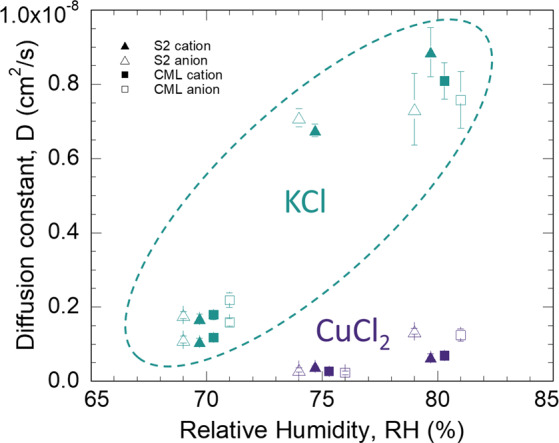
Calculated K, Cu, and Cl diffusion constants (*D*) as a function of RH. For clarity, the S2 secondary cell wall and compound middle lamella (CML) *D* are offset to the negative and positive sides, respectively, for a given RH. The dashed oval bounds the KCl results. The CuCl_2_ are outside the dashed oval. The error bars represent uncertainties based on a least squares analysis for corresponding straight-line fits in Fig. 4.

The calculated diffusion coefficients for each salt ion in the S2 and CML are shown in Fig. 5. For a given salt, both the S2 and CML exhibited similar *D* values that increased with RH. Additionally, the Cl anions diffused at similar rates as the associated salt cation, indicating that the ions diffused together to maintain charge neutrality. In a water-swollen polymer, salt ions are generally dissociated and move as ions solvated primarily by water and, to a lesser extent, polymer segments^68^. The rate of diffusion for an ion is approximately inversely proportional to its effective size, which is determined by the ion and its solvating water. Although interactions between ions and polymer segments can complicate matters, relative ion diffusion rates in water-swollen polymers can be approximated using their hydrated radii, which is greater for Cu^+2^ (4.19 Å) than K^+^ (3.31 Å) or Cl^−^ (3.32 Å)^69^. Additionally, the electrostatic attraction of the divalent Cu^+2^ to the wood polymer functional groups that serve as complexation sites is stronger than that of the monovalent K^+^ ^70^.

**Figure 6:**
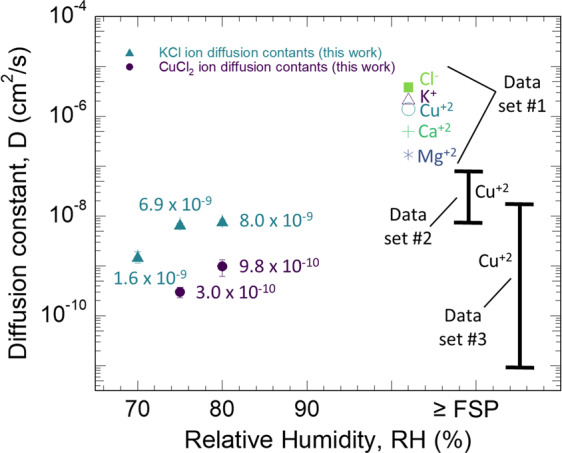
Comparison of average KCl and CuCl_2_ ion diffusion constants (*D*) measured in this study to data sets reported in the literature assessed from experiments at or above the fiber saturation point (FSP). Error bars for *D* measured in this study are one standard deviation. Data set #1 is calculated from the ionic conductances measured by Simons and coworkers^58,59,71^. Data set #2 is from Jeremic and coworkers^56^ who analyzed the time-dependent progression of an ion front reported by Yata and coworkers^57^ to estimate Cu ion *D* in the longitudinal direction of wood cell walls to be in the range of 0.8 × 10^−8^ to 8.6 × 10^−8^ cm^2^/s. Data set #3 is from Cooper^55^ who calculated values of Cu ion *D* to be between 1 × 10^−11^ and 1.7 × 10^−8^ cm^2^/s depending on wood species and treatment conditions.

The averaged ion diffusion constants from a given salt are plotted in Fig. 6. Compared to the ions from KCl experiments, the CuCl_2_ ions diffused by a factor of approximately 20 slower at 75% RH and a factor of 10 slower at 80% RH. The amount of moisture in the wood sections over the 70 to 80% RH conditions was estimated to be 12% to 15% moisture content based on a literature absorption isotherm for southern pine^26^. The moisture contents were taken from the absorption isotherm because the sections were initially conditioned under dry nitrogen before the RH was stepped up to the experimental RH value. For comparison, ion diffusion constants reported in the literature for wood at or above FSP are also plotted in Fig. 6. The literature data show a large amount of scattering, likely because of the difficulty of measuring diffusion in wood cell walls. However, useful comparisons can still be made within a set of data. For example, consider the diffusion constants from data set #1 from the experiments of Simons and coworkers^58,59,71^. In this data set, the divalent cations were slower than the monovalent cations under water-saturated conditions, similar to the *D* measurements at the lower moisture conditions in this study.

## Discussion

To check the accuracy of the diffusion constant measurements and better understand electrical conductivity mechanisms in wood, comparisons were made to S2 and CCML electrical conductivity measurements. Most experimental evidence agrees that electrical conductivity in wood is ionic^53,72,73^. Furthermore, over the 70–80% RH range studied here and at higher levels of moisture, there is convincing evidence that in unmodified wood the electrical charge is carried by the endogenous mineral ions^59,71,73,74^, of which K^+^, Ca^2+^, and Mg^2+^ are the most abundant^75,76^. Based on the much larger K^+^ *D* calculated as compared to the divalent cations in this work (Fig. 5) and for the literature data set #1 in Fig. 6^58,59,71^, we assert that K^+^ is most likely to control the electrical conductivity of wood because it is the most mobile of the most abundant inorganic ions. Therefore, we calculated the ion conductivity σ using the average KCl diffusion constants in Fig. 6 for the K^+^ *D* and the Nernst-Einstein relationship

2 $$\sigma =\frac{DN{q}^{2}}{kT}$$

where *N* is the number of ion carriers, *q* is the ion charge of K^+^ (1.6 × 10^−19^ C), *k* is Boltzmann’s constant (1.38 × 10^−23^ J/K), and *T* is temperature (298 K). The value for *N* was estimated using the average value for K C_o_ measured in the KCl samples, which was 6 ± 2 mol/m^3^, or 3.6 ± 1.2 × 10^24^ K atoms/m^3^, for all S2 and CML (Fig. 3a). This concentration of K agreed relatively well with the values of 2 × 10^24^ atoms/m^3^ obtained from the XFM maps of unmodified transverse loblolly pine sections in previous work^61^. Furthermore, the XFM maps of the transverse plane in the previous work showed similar K concentrations between the S2, CML, and CCML cell wall layers^61^.

**Figure 7:**
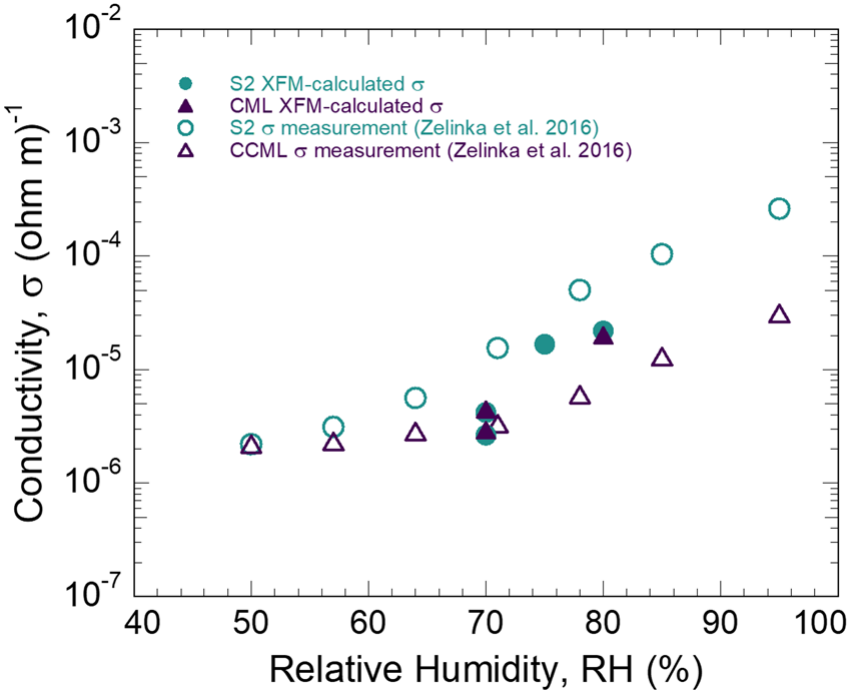
Comparisons of XFM-calculated ion conductivity (σ) for the S2 secondary cell wall layer and compound middle lamella (CML) using the Nearnst-Einstein relationship (Eq. 2) and average RH-dependent KCl diffusion constants (Fig. 6). Also included are σ measurements in the S2 and corner CML (CCML) from Zelinka and coworkers^63^.

The XFM-calculated σ are plotted in Fig. 7 as a function of RH. Also plotted in Fig. 7 are σ recently measured in the S2 and CCML of loblolly pine^63^. The magnitudes of the calculated and measured σ overlap, which further confirms that over these moisture conditions σ is controlled by K^+^ diffusion. The similar magnitudes also support the accuracy of our diffusion constant measurements.

The σ comparison in Fig. 7 was also found useful for better understanding ionic conductivity through S2 and CML. To a close approximation, the increase of XFM-calculated σ over the 70%-80% RH range matched that of the electrical conductivity measurements. This indicates that the ion conductivity at these levels of moisture increases proportionally with ion mobility. This contradicts previous models asserting that ion mobility remains unchanged with increasing moisture, and the increase in conductivity is a result of ion dissociation causing an increase in charge carriers^52,53^.

A difference between calculated and measured σ is that the S2 measured σ was substantially higher than the CCML measured σ. In contrast, we obtained the same *D*, and therefore the same calculated σ, for both S2 and CML. It was possible that the CCML, in which the σ was measured^63^, had a different structure and composition than the CML, from which the diffusion rates were assessed in this study. The CCML is reported to have a higher percentage of lignin than the CML^77^. We may also have measured the same *D* for S2 and CML because the CML is so thin and movement from S2 to CML could have occurred faster than the progression of the primary concentration gradient in the CML.

These results, along with recent cell wall nanomechanical spectroscopy results^35^, collectively provide further support for the proposed mechanism that at moisture levels at and above about 55% RH, chemical species such as inorganic ions diffuse through wood cell walls via interconnecting regions of rubbery amorphous polysaccharides^12^. This mechanism is contradictory to previous assertions that ions diffusion through wood cell walls is an aqueous process occurring through water pathways^50–54^. This new understanding of diffusion will enable lignocellulosic biomass researchers to apply established polymer engineering approaches to co-optimize processing conditions and the molecular architecture of lignocellulosic materials to control diffusion for specific end uses.

For biorefinery applications, promoting diffusion is most likely beneficial. One potential avenue could be pursued by molecular biologists who can now design plants to make cell walls with specified molecular structures, such as the organization of glucuronic acid substituents on xylan^78^. The hemicelluloses side groups could be modified such that they pack more “loosely”, so water can more easily plasticize them to create free volume and push the polymers through their glass transition. Additionally, it is well known that solvents other than water can be more effective at swelling wood^79^, and presumably producing more free volume that could also facilitate diffusion.

In contrast, with regards to wood degradation mechanisms such as decay and fastener corrosion, preventing cell wall diffusion may be desirable because it would be an effective way to protect wood. The simplest and most effective option would be to keep wood dry. Without moisture, wood polymers would be expected to remain in their glassy state^25^ and the free volume needed for the diffusion that facilitates degradation would not exist. However, increased utilization of wood requires improving it so it can be used in higher moisture environments. This could be achieved by preventing diffusion through either minimizing water sorption that leads to plasticization or breaking up the continuous rubbery amorphous polysaccharide diffusion pathways. Again, molecular biologists may be able to modify side group substituents on hemicelluloses such that they pack more “tightly” and make it more difficult for water molecules to be absorbed and cause the glass transition. Another option would be to chemically modify the wood polymers, such as the recently developed PolyCatNap treatment for archaeological wood conservation^80^. Another chemical treatment is acetylation, in which hydroxyl groups are replaced with bulky, less hydrophilic acetyl groups. Indeed, recent XFM experiments on acetylated wood cell walls found that the RH threshold for ion diffusion increased with increasing levels of acetylation^64^.

## Conclusions

An XFM-based technique was developed to measure diffusion constants of ions in individual wood S2 and CML as a function of RH conditioning. Diffusion was observed to be Fickian. Measured diffusion constants of ions locally applied using KCl or CuCl_2_ salt solutions varied from 3.0 × 10^−10^ to 8.0 × 10^−9^ cm^2^/s. Diffusion rates increased with increasing RH. For a given salt, the Cl anion diffused at the same rate as the salt cation, indicating charge neutrality was being maintained during diffusion. The KCl ions diffused faster than CuCl_2_ ions, as expected based on the larger size of the Cu^2+^ hydrated ion complex. To within the accuracy of these measurements, no differences between diffusion constants in the S2 and CML were observed for a given salt solution and RH. Using the Nernst-Einstein relationship and RH-dependent KCl diffusion constants, the cell wall σ was calculated and compared to experimentally measured values from literature. Our XFM-derived σ values were in good agreement with the conductivity-based σ values, further validating that σ is ionic and that, at least over the moisture conditions tested, σ is controlled by ionic mobility. Finally, the results further support that inorganic ions transport through wood cell wall layers via interconnecting regions of rubbery amorphous polysaccharides and not the previously proposed water pathways. With this new insight, researchers can now utilize the fundamental frameworks for diffusion in the polymer science literature to more efficiently design the molecular architecture of lignocellulosic cell walls with diffusion properties for specific applications.

## Materials and Methods

All thin 2–μm–thick sections used in the experiments were cut from the same mature, compression-wood-free latewood growth ring of commercial kiln-dried loblolly pine (*Pinus taeda*) using a diamond knife fit into a Leica EM UC7 ultramicrotome (Wetzlar, Germany). The sections were cut in the tangential-longitudinal orientation and measured about 200 µm in the tangential direction and 2 mm in the longitudinal direction. Sample holders consisted of 0.13-mm-thick Kapton (DuPont, Wilmington, Delaware, USA) film with a 1-mm-wide slot. The sections were mounted across the slot using small pieces of Kapton tape to secure each end. Ions were locally applied using 0.5 M aqueous solutions of either KCl or CuCl_2_. Micro droplets of a solution were deposited manually to chosen cell walls using a sharpened piece of polystyrene foam under a dissecting microscope. The light from the dissecting microscope created heat that caused the water in the micro droplet to evaporate within about 1 s, resulting in an applied ion front in the cell walls.

For XFM mapping, a sample holder was secured inside a custom RH chamber built for beamline 2-ID-E at the Advanced Photon Source at Argonne National Laboratory (Argonne, IL, USA). The chamber utilized the beamline kinematic mount and was constructed of an aluminum frame covered by Kapton film^34^. The RH inside the chamber was controlled by a HumidiSys RH generator (Instruquest, Coconut Creek, FL) supplied with nitrogen gas. The temperature and RH were measured inside the chamber using a Sensirion (Staefa, Switzerland) SHT1x sensor. The measured temperature during all experiments was 33 °C. The incident X-ray beam energy was 10.2 keV, and the X-ray spot size was approximately 0.5 µm in diameter. XFM maps were built by raster scanning the X-ray probe over the section using 5-ms dwell times, horizontal step sizes that ranged from 0.3 to 0.6 μm, and vertical step sizes that ranged from 0.3 to 1 μm. The step sizes were constant for a given XFM map but varied between experiments to control the length of time needed to complete a scan. Each map took between 8 and 20 minutes to complete. Initially, XFM mapping was performed under a dry nitrogen purge to locate the applied cell wall and obtain an initial map of the ion front. Then the RH was raised to the desired level. The chamber took about three minutes to reach the target RH, after which time-lapse XFM mapping was initiated and successive ion maps were captured. Based on how quickly the wood section was observed to wet and dry during the application of the aqueous salt micro droplet, the thin section is expected to equilibrate nearly instantaneously with RH changes in the humidity chamber.

The quantified elemental maps were created using the MAPS software package^81^ in which full spectra were fitted to modified Gaussian peaks, the background was iteratively calculated and subtracted, and the results were compared to standard reference materials (RF4-100-S1749, AXO DRESDEN GmbH, Heidenau, Germany). All fluorescing elements were mapped, but only relevant elements are discussed. Image analysis and presentation were carried out using FIJI^82^ and 32-bit tiff images exported from MAPS. The image pixel size was converted to 0.3 by 0.3 µm using a bilinear interpolation. Additionally, a geometric correction factor was applied to the horizontal direction because the sample holders were mounted at a 15° angle with respect to the direction of horizontal stage motor.

To extract ion intensity profiles along the diffusing direction, line segments were first drawn following the CML in the 0 min Ca map. The line segments were copied to each successive Ca map in the time series, and slight modifications were made to ensure that the line followed the same segment of the CML. Lines were then copied to the corresponding salt ion map, and the intensity profiles were extracted from the CML using a line with a 0.9 µm (3 pixel) width (red lines following CML in Fig. 2e–h,k–n). To extract profiles from the S2, for a given Ca map the CML line was moved vertically until centered between the CML and lumen edge. Then the line was copied to the corresponding salt ion map and its width increased to 3.6 µm (12 pixels) (broad semi-transparent white lines in Figs. 2e–h,k–n) to extract the intensity profile. For the KCl 75% RH specimen, the XFM maps did not include enough of the CML to draw a complete line. Therefore, the interface of the wood cell wall and lumen was used instead of the CML to draw and align the line segments used to extract S2 intensity profiles.

## Supplementary information

Supplementary Information.

## Data Availability

The data that support the findings of this study are available from the corresponding author, JEJ, upon reasonable request.
