# Supplementary material for: Measurement of moisture-dependent ion diffusion constants in wood cell wall layers using time-lapse micro X-ray fluorescence microscopy
Source: Sci Rep. 2020 Jun 18;10:9919. doi: 10.1038/s41598-020-66916-8 (PMC7303177; doi:10.1038/s41598-020-66916-8)
Supplement: Supplementary file 1 — Supplementary Information. [file 41598_2020_66916_MOESM1_ESM.pdf]

Measurement of moisture-dependent ion diffusion constants in wood cell wall layers using time-lapse micro X-ray fluorescence microscopy

Joseph E. Jakes[1]*, Samuel L. Zelinka[2], Christopher G. Hunt[1], Peter Ciesielski[3], Charles R. Frihart[1], Daniel Yelle[1], Leandro Passarini[2], Sophie-Charlotte Gleber[4], David Vine[4], and Stefan Vogt[4]

[1]Forest Biopolymers Science and Engineering, USDA Forest Service, Forest Products Laboratory, One Gifford Pinchot Drive, Madison, WI 53726.

[2]Building and Fire Sciences, USDA Forest Service, Forest Products Laboratory, One Gifford Pinchot Drive, Madison, WI 53726.

[3]Biosciences Center, National Renewable Energy Laboratory, 15013 Denver W Pkwy, Golden, CO 80401

[4]X-ray Science Division, Advanced Photon Source, Argonne National Laboratory, 9700 S. Cass Avenue, Lemont, IL 60439.

*corresponding author: Joseph E. Jakes; Address: One Gifford Pinchot Drive, Madison, WI 53726; Phone number: 1-608-231-9308; E-mail: joseph.e.jakes@usda.gov

<u>Supplementary Information</u>

Although both infinite and finite source diffusion were observed in the experiments (Figure 3), here it will be demonstrated how a single analysis can be used to analyze both. First, it was assumed that diffusion constant *D* was a constant and diffusion followed Fick's second law for one-dimensional diffusion

$$\frac{\partial C(x,t)}{\partial t} = D\frac{\partial^2 C}{\partial x^2} \qquad \text{(S1)}$$

The one-dimensional assumption is valid because the experiments were performed on thin sections in which diffusion could not occur into the air above or below the section, and the observation that diffusion proceeded with a straight-line front that was perpendicular to the diffusion direction. For infinite source diffusion, $C(x)$ at $t = 0$ is assumed to be a step function at $x = 0$ with the applied concentration C_s for $x < 0$ and background concentration C_o for $x > 0$, shown by red line in Figure S1a. The C for $x < 0$ remains constant for $t \geq 0$. For these infinite source boundary conditions, a solution for Equation S1 for $t > 0$ is given by

$$C(x,t) = C_s - (C_s - C_o)\left[\operatorname{erf}\left(\frac{x}{\sqrt{4Dt}}\right)\right] \quad \text{(S2)}$$

where erf is the error function. For finite source diffusion, $C(x)$ at $t = 0$ can also be assumed to be a step function at $x = 0$ with $C(x) = C_s$ for $x < 0$ and $C(x) = C_o$ for $x > 0$, shown by red line in Figure S1b. However, in this case, C for $x < 0$ does not remain constant for $t > 0$. For these finite source boundary conditions, a solution for Equation S1 is

$$C(x,t) = C_o + \frac{1}{2}(C_s - C_o)\left[1 - \operatorname{erf}\left(\frac{x}{\sqrt{4Dt}}\right)\right] \quad \text{(S3)}$$

Figure S1 illustrates ideal concentration profiles, denoted $C(x,t)$, for both infinite and finite source diffusion. For this illustration, C_s and C_o were chosen to show that $C(x,t)$ for $x > 0$ and $t > 0$ can be the same. Therefore, a single analysis could be used to analyze the S2 secondary cell wall (S2) and compound middle lamella (CML) $C(x,t)$ for $x > 0$, regardless of whether the experiments better fit the boundary conditions for infinite or finite source diffusion.

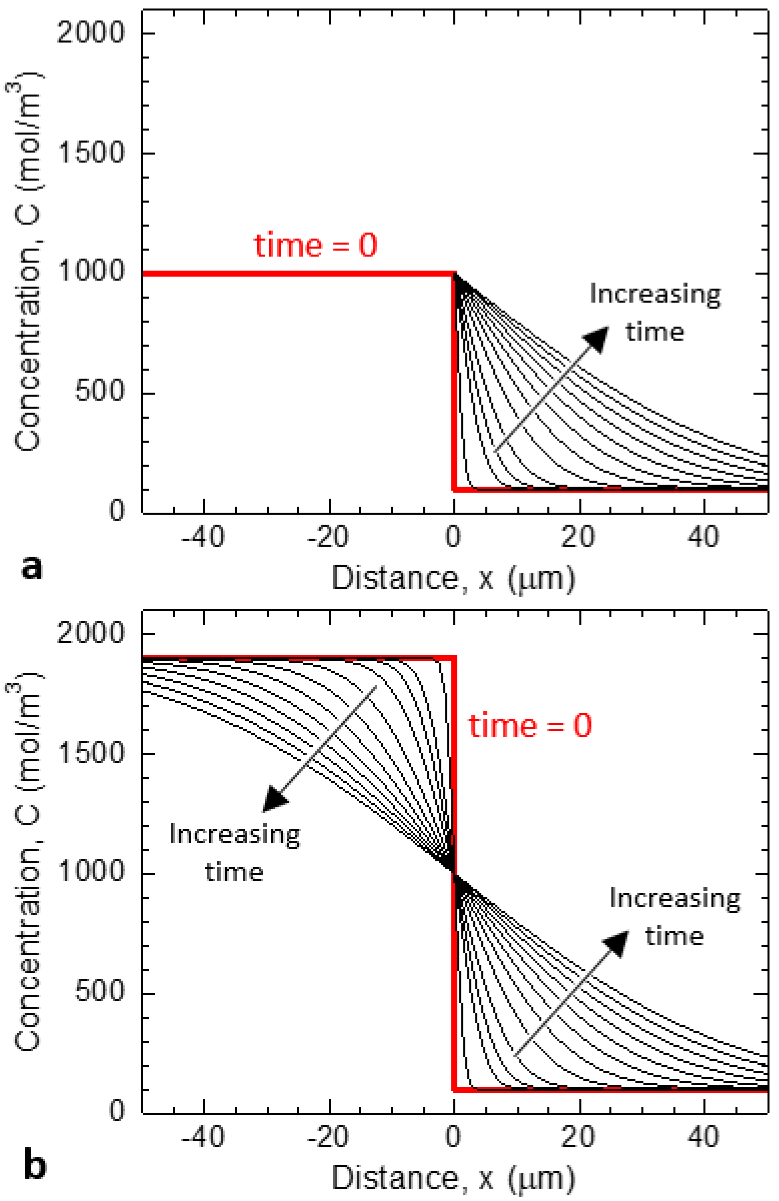

<u>Figure S1:</u> Concentration profiles for a) infinite source diffusion (Equation S2) and b) finite source diffusion (Equation S3). Red lines indicate step function concentration profiles at time = 0. For the infinite source, C_s = 1000 mol/m^3 and C_o = 100 mol/m^3. For the finite source diffusion, C_s = 1900 mol/m^3 and C_o = 100 mol/m^3.

In this work, the experimental $C(x,t)$ data were analyzed using an analytical model based on the solution for infinite source diffusion in Equation S3. The C_s was defined as the average value of C for the concentration profiles at x = 0. The value of C_o was defined from the initial

concentration profile far from the applied ions. In the $C(x,t)$ data of Figure 2 in the manuscript, the values of C_s and C_o are illustrated. The final parameter to determine is t, which could not be directly defined because the $C(x,t = 0)$ profiles did not adequately resemble the step function assumed in the boundary conditions. To overcome this problem, an analysis presented by Porter and Easterling[1] was utilized. It was first noted that erf(0.5) ≈ 0.5. Therefore, at $C(x) = 0.5*(C_s + C_o)$ it can be shown using Equation S3 that

$$0.5 = \frac{x_{\mathrm{h}}}{\sqrt{4Dt}} \qquad \text{(S4)}$$

where x_h is termed the "half-distance" and is the x that satisfies $C(x) = 0.5*(C_s + C_o)$ for a given $C(x)$ profile. Equation S4 can be rearranged and squared to give

$$x_{\mathrm{h}}^2 = Dt \qquad \text{(S5)}$$

We then assumed the initial concentration profile to be representative of $C(x,t_o)$, where t_o was an unknown time that accounted for the effective time it would have taken for a step function to become the $C(x,t = 0)$ profile. Then, $t = t_i+t_o$ was substituted into Equation S5, where t_i was the time after the initial XFM map. This resulted in the relationship

$$x_{\mathrm{h}}^2 = D(t_i + t_o) = Dt_i + Dt_o \qquad \text{(S7)}$$

In our experiments, t_i was known for each $C(x)$. Therefore, a plot of x_h^2 vs. t_i could be made. If our proposed analysis based on Fick's second law is valid, then the plot of experimental data shown in figure 4 will form a straight line of slope D.

<u>References</u>

1. Porter, D. & Easterling, K. Phase Transformations in Metals and Alloys. (Nelson Thornes Ltd, 1992).
